# Microbial shifts in the porcine distal gut in response to diets supplemented with *Enterococcus Faecalis* as alternatives to antibiotics

**DOI:** 10.1038/srep41395

**Published:** 2017-02-06

**Authors:** Pinghua Li, Qing Niu, Qingtian Wei, Yeqiu Zhang, Xiang Ma, Sung Woo Kim, Mingxin Lin, Ruihua Huang

**Affiliations:** 1 Institute of Swine Science, Nanjing Agricultural University, Nanjing, 210095, China; 2 Huaian Academy of Nanjing Agricultural University, Huaian, 223005, China; 3 Department of Animal Science, North Carolina State University, Raleigh, North Carolina, 27695, United States of America; 4 Changxing Ecoagriculture Co. Limited, Yixing, 214246, China

## Abstract

Gut microbiota plays an important role in host health and nutrient digestion of animals. Probiotics have become one of effective alternatives to antibiotics enhancing animal health and performance through modulating gut microbiota. Previously, our research demonstrated that dietary *Enterococcus Faecalis* UC-100 substituting antibiotics enhanced growth and health of weaned pigs. To investigate the alterations of microbiota in the distal gut of pigs fed *E. faecalis* UC-100 substituting antibiotics, this study assessed fecal microbiota in pigs from different dietary treatments: the basal diet group, the *E. faecalis* group, and the antibiotic group on d 0, 14, and 28 of feeding through 16 S rRNA sequencing. Twenty-one phyla and 137 genera were shared by all pigs, whereas 12 genera were uniquely identified in the *E. faecalis* group on d 14 and 28. Bacterial abundance and diversity in the *E. faecalis* group, bacterial diversity in the antibiotic group, especially abundances of *Fibrobacteres* phylum and 12 genera in the *E. faecalis* group and antibiotics group were lower than that in the basal diet group on d 28. These results showed that microbial shifts in the porcine gut in response to diets containing *E. faecalis* were similar to the response to which containing antibiotics.

The large and diverse gut microbiota plays an important role in nutrient digestion and health of the host^1^,^2^,^3^,^4^. Weaning process always accompanies changes in intestinal structure and gut micro-ecosystem causing digestion dysfunction, diarrhea and growth inhibition in weaned pigs. Antibiotics have been used in pig production especially for weaned pigs to treat infectious diseases as well as to promote growth for more than 50 years^4^. However, the excessive use of antibiotics has caused the emergence of resistance of pathogenic bacteria to antimicrobials and a direct threat to human and animal health^5^,^6^,^7^. Therefore, efforts to find alternatives to antibiotics are being implemented to preserve the efficacy of current antimicrobials.

Probiotics and their metabolites have been suggested as the most desirable alternatives to support animal health, and as an effective way to promote growth through modulating gut microbiota in livestock^2^,^8^,^9^,^10^. Seeking effective probiotic bacteria as an alternative to antibiotics and untangling how these probiotics might affect host intestinal microbiota and immunity to improve the health and performance are essential steps for the successful application of probiotics in pig production^11^.

*Enterococcus faecalis (E. faecalis*), a lactic acid bacterium and common inhabitant in the gut^12^,^13^, is one of the most common species of *Enterococci. Enterococci* species including *Enterococcus faecium* strains have been studied for possible use as a probiotic and have shown beneficial effects on the health in pigs^14^,^15^,^16^. Some *E. faecalis* strains have been found beneficial to mice and humans improving their immunity^17^,^18^ and inhibiting pathogenic infection by producing bacteriocin^19^,^20^,^21^,^22^. However, limited studies are available demonstrating the application of the *E. faecalis* strains as alternatives to antibiotics in pig production^23^,^24^.

Previously, our research^25^ showed that weaned pigs fed a diet supplemented with *E. faecalis* UC-100 (200 g/t, ≥ 1 × 10^10^ CFU/g) had an increased body weight gain (increased by 30.9% on d 28 of feeding) and a decreased feed to gain ratio (decreased by 9.2% on d 28 of feeding) and incidence of diarrhea (decreased by 58.3% on d 28 of feeding) compared with weaned pigs fed a basal diet, whereas no difference was observed compared with weaned pigs fed a diet supplemented with antibiotics (bacitracin zinc: 40 g/t, aureomycin: 75 g/t and colistin: 20 g/t). However, specific mechanisms of the action for *E. faecalis* UC-100 were not clear. Considering that gut microbiota plays an important role in nutrient digestion and health of the host animals^1^,^2^,^3^,^4^, and many probiotics can promote animal growth and health through modulating gut microbiota^2^,^8^,^9^,^10^, it is hypothesized that *E. faecalis* UC-100 exerts growth and health promoting effect by altering gut microbiota in pigs. The objective of this study was to investigate alterations of microbiota in the porcine distal gut in response to dietary treatment of *E. faecalis* UC-100 as alternatives to antibiotics.

## Results

### DNA sequence data and quality control

A total of 2,355,992 paired-end 250-bp reads were acquired. The total read length was 1.97 gigabases (GB), and the average read length per sample was 0.05 GB. On d 0, 14, and 28 of feeding, there were 201,303, 212,206, and 222,669 raw reads in pigs from the basal diet group; 222,402, 217,627, and 216,349 raw reads in pigs from the *E. faecalis* group; 196,387, 207,015, and 215,410 raw reads in pigs from the antibiotic group, respectively (Table S1).

After quality control, 1,846,755 high quality sequences were obtained. On average, 51,298 sequences were obtained per sample. On d 0, 14, and 28 of feeding, there were 6,073, 6,378, and 6,908 operational taxonomic units (OTUs) in pigs from the basal diet group; 6,032, 6,459, and 6,235 OTUs in pigs from the *E. faecalis* group; 5,446, 5,856, and 6,274 OTUs in pigs from the antibiotic group, respectively, based on 97% species similarity (Table S1). A total of 9,966 OTUs were identified from all fecal samples (Table S1). Most OTUs were shared among groups at the same age, only 953 and 749 OTUs were uniquely identified in pigs from the *E. faecalis* group on d 14 and 28 of feeding, respectively (Fig. S1A). In addition, 753 and 729 OTUs were uniquely identified in pigs from the antibiotic group on d 14 and 28 of feeding, respectively (Fig. S1B).

### Shifts in microbial abundance and diversity after *E. faecalis* treatment

**Figure 1:**
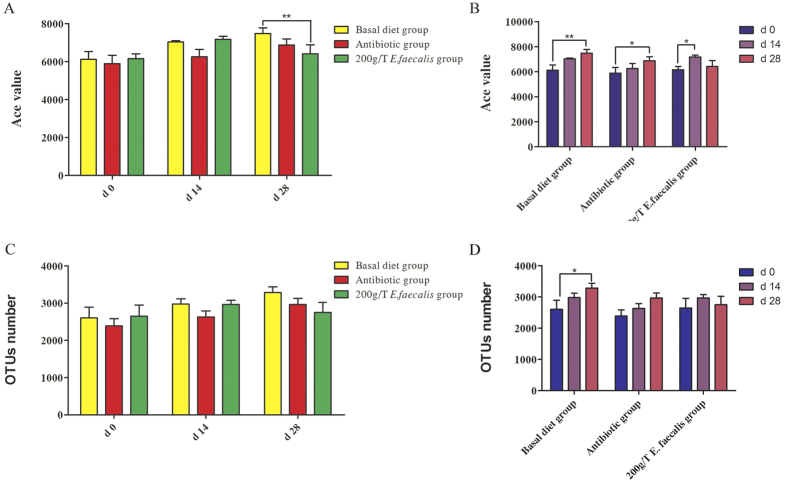
Comparison of the Ace index and OTUs in the nine groups. The number of observed OTUs sharing ≥ 97% nucleotide sequence identity. Bacterial abundance was reflected with Ace index and bacterial diversity was reflected with OTUs number. (**A**) Ace index was compared among the basal diet group, the *Enterococcus faecalis* group and the antibiotic group at 3 different phases (d 0, 14, and 28 of feeding), respectively. (**B**) Ace index was compared among 3 different phases in the basal diet group, the *Enterococcus faecalis* group and the antibiotic group, respectively. (**C**) OTUs number was compared among the basal diet group, the *Enterococcus faecalis* group and the antibiotic group at 3 different phases, respectively. (**D**) OTUs number was compared among 3 different phases in the basal diet group, the *Enterococcus faecalis* group and the antibiotic group, respectively. (*p < 0.05, **p < 0.01).

**Figure 2:**
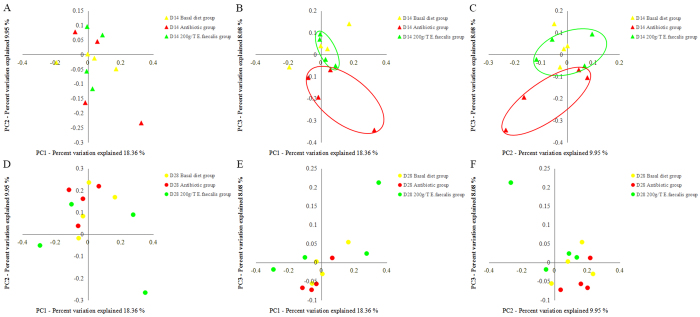
PCoA of the weighted UniFrac distances for three groups on d 14 (**A,B** and **C**) and d 28(**D,E** and **F**) of feeding. The percent variation explained by each principal coordinate (**A,B,C,D,E** and **F**) is indicated on the axes.

Good’s coverage was at least 96% for each group. The range of the calculated value for Ace value was 5,887-7,482 over the 3 sampling times (d 0, 14, and 28 of feeding). On d 28 of feeding, the bacterial abundance in pigs from the *E. faecalis* group was lower (*P* < 0.01) than those from the basal diet group, whereas there was no difference between the *E. faecalis* group and the antibiotic group (Fig. 1A). And no difference in bacterial abundance was detected between the antibiotic group and the basal diet group on either d 14 or 28 of feeding (Fig. 1A). Compared with d 0 of feeding, bacterial abundance in pigs from the basal diet group and the antibiotic group increased (*P* < 0.01) on d 28, whereas bacterial abundance in the *E. faecalis* group increased (*P* < 0.05) on d 14 (Fig. 1B). No difference in bacterial diversity was detected among 3 groups on either d 14 or 28 of feeding (Fig. 1C). Compared with d 0 of feeding, bacterial diversity increased (*P* < 0.05) on d 28 from the basal diet group, whereas no change in the *E. faecalis* group and the antibiotic group (Fig. 1D). Weighted UniFrac distances were used to estimate β-diversity and to compare the three diet groups on d 14 (Fig. 2A,B and C) and d 28 of feeding (Fig. 2D,E and F). The PCoA plot of the weighted UniFrac distances showed that the three diet groups did not form distinct clusters on either d 14 or 28 of feeding, although the antibiotic group microbiota tended (*P* = 0.063) to separate from the *Enterococcus faecalis* group microbiota along principal coordinate 3 on d 14 (Fig. 2B and C).

### Shifts in community membership after *E. faecalis* treatment

**Figure 3:**
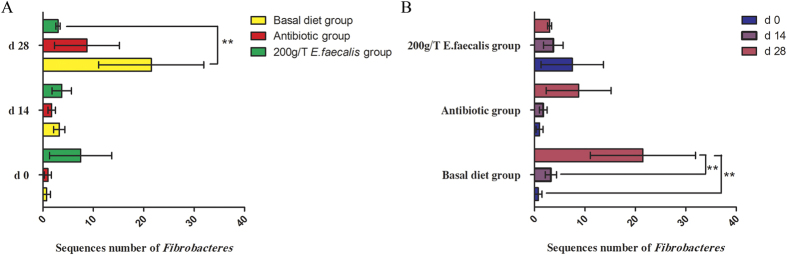
The bacterial abundances of *Fibrobacteres* significantly differ among different groups. (**A**) The abundances of *Fibrobacteres* was compared among 3 different treatments on d 0, 14, and 28 of feeding, respectively. (**B**) The abundances of *Fibrobacteres* was compared among 3 different phases of experiment in the basal diet group, the *Enterococcus faecalis* group and the antibiotic group, respectively.

A total of 21 phyla were shared by pigs from all groups (Fig. S2 A), as follows: *Actinobacteria, Bacteroidetes, Caldiserica, Chlamydiae, Chloroflexi, Crenarchaeota, Cyanobacteria, Deferribacteres, Euryarchaeota, Fibrobacteres, Firmicutes, Fusobacteria, Lentisphaerae, Planctomycetes, Proteobacteria, Spirochaetes, Synergistetes, Tenericutes, Thermi, TM7*, and *Verrucomicrobia*. Of them, *Firmicutes* was the most dominant among the 21 phyla (*P* < 0.01) in the samples, and comprised more than 85% of the total sequences. *Tenericutes* and *Bacteroidetes* were the 2nd and 3rd dominant phyla which comprised only 1% of the total sequences. The bacterial abundance of *Fibrobacteres* in pigs from the *E. faecalis* group was lower (*P* < 0.01) than that in the basal diet group on d 28 of feeding, while no difference in bacterial abundance of *Fibrobacteres* was detected between the *E. faecalis* group and the antibiotic group (Fig. 3A,B). Meanwhile, the bacterial abundance of *Fibrobacteres* in pigs from the antibiotic group tended (*P* = 0.063) to be lower than that in the basal diet group on d 28 of feeding (Fig. 3A).

At the genus level, a total of 137 genera were identified from all samples (Fig. S2 B). The 11 most abundant genera, containing more than 85% of the total sequences, were *Lactobacillus, Bulleidia, Clostridium, Streptococcus, Chlamydia, Coprococcus, Oscillospira, Eubacterium, Treponema, Ruminococcus, and Blautia*. Of them, *Chlamydia* was a member of the phylum *Chlamydiae, Treponema* belongs to the phylum *Spirochaetes* and the other 9 genera belong to the phylum *Firmicutes*. Among the 11 most abundant genera, *Lactobacillus, Bulleidia,* and *Clostridium* were the most predominant genera, accounting for 23, 13, and 11% of total sequences, respectively. Most genera were shared among 3 groups at the same age, only 9 genera (*Anaerovorax, Brevibacterium, Deinococcus, Facklamia, Ignatzschineria, Mycoplasma, Pedobacter, Sphingobium* and *Vibrio*) and 3 genera (*Burkholderia, Paraprevotella* and *Stenotrophomonas*) were uniquely identified in pigs from the *E. faecalis* group on d 14 and 28 of feeding, respectively (Table 1). Only 10 (*Acetobacter, Aequorivita, Anaerococcus, B-42, Holdemania, HTCC, Mycobacterium, Sporanaerobacter, Tsukamurella* and *Veillonella*) and 1 (*Ramlibacter*) genera were uniquely identified in pigs from the antibiotic group on d 14 and 28 of feeding, respectively (Table 2).

The abundance of 12 genera (*Acholeplasma*, Arcobacter, *Caldicoprobacter, Desulfotomaculum, Ignatzschineria, KSA1, Leptolyngbya, Natronincola_Anaerovirgula, Pseudomonas, Pseudoramibacter_Eubacterium, Tepidimicrobium*, and *Tissierella_Soehngenia*) in pigs from both the *E. faecalis* group and the antibiotic group were lower (*P* < 0.05) than that in the basal diet group on d 28 of feeding, while no difference in bacterial abundance of these genera were detected between the *E. faecalis* group and the antibiotic group (Tables 3,4). The abundance of 5 genera (*Anaerococcus, Fibrobacter, Megasphaera, Selenomonas* and *Sharpea*) changed (*P* < 0.05) in pigs from the *E. faecalis* group than that in the basal diet group, and the abundance of 4 genera (*Bacillus, Sphaerochaeta, Vibrio* and *Zhouia*) changed (*P* < 0.05) in pigs from the antibiotic group than that in the basal diet group on d 28 (Table 5). However, no difference in bacterial abundance of these 9 genera was detected between the *E. faecalis* group and the antibiotic group (Table 5).

## Discussion

Dietary supplementation of *E. faecalis* strains, as a probiotic, has become one of effective alternatives to the use of antibiotics to increase health and growth performance of pigs^24^,^25^ as it has been shown that probiotics can affect gut microbiota which plays an important role in health and nutrient digestion in pigs^1^,^2^,^3^,^4^. Although many studies have examined the impact of antibiotics on the gut microbiota in pigs^1^,^4^,^26^,^27^,^28^,^29^, there is very little information on how consumed *E. faecalis* affect the entire porcine gut microbiota^24^,^30^. Because many *E. faecalis* strains can inhibit pathogen by producing bacteriocin^19^,^20^,^21^,^22^, we try to study if *E. faecalis* UC-100 administration could induce alteration of the gut microflora to directly or indirectly impact porcine health and performance. Meanwhile, it is important to explore if *E. faecalis* UC-100 administration could cause alteration of the composition or activity of the host normal microbiota to exclude the possibility of the occurrence of undesirable microbiota changes before we applicate the *E. faecalis* UC-100 in pig production.

Since dietary supplementation of 200 g/t *E. faecalis* UC-100 showed the benefits similar to antibiotics supplementation from the previous manuscript^25^, samples from the *E. faecalis* UC-100 group(200 g/t), the basal diet group and the positive control diet group collected on d 0, 14, and 28 of feeding were used to determine alteration of the distal gut microbiota population in response to the treatment with *E. faecalis* substitute for antibiotics. We obtained 1,846,755 high-quality sequences from all samples, and the read counts were greater than those in previous studies in pigs^1^,^4^,^28^. Moreover, according to Good’s coverage index (96%) of each sample, the modified sequences were comprehensively enough to cover most bacterial diversity.

Venn diagrams were generated to make qualitative comparisons among *E. faecalis* group, antibiotic group and basal diet group at the same age. Most of the OTUs were shared between groups at the same age (Fig. S1A), which indicates that unique OTUs to each group were more likely to be found as less abundant OTUs, and this result is consistent with previous study^1^. In the basal diet group, bacterial abundance and diversity were increased with age, and these results are in accordance with a previous study^31^. Whereas, the increase of bacterial diversity and abundance were inhibited in pigs from the *E. faecalis* group compared with the basal diet group on d 28 of feeding. Mechanisms whereby *E. faecalis* UC-100 decrease bacterial diversity and abundance may be that *E. faecalis* strains can produce bacteriocin to inhibit pathogen and modulate other gut microbiota^19^,^20^,^21^,^22^. Although the increase of bacterial diversity was inhibited in pigs from the antibiotic group compared with the basal diet group on d 28 of feeding, no difference in bacterial abundance and diversity was detected between the antibiotic group and the basal diet group, and the addition of antibiotic to the swine diet did not shift the overall microbial community structure (*β*-diversity indices) on either d 14 or 28 of feeding. These results were similar to a previous study which showed that overall microbial community structure, microbial abundance and diversity in weaned pigs were not affected by chlortetracycline treatment^28^. Poole *et al*.^32^ also found no significant effect on *a*-diversity when pigs were fed with chlortetracycline for 28 days. In contrast, Looft *et al*.^33^ observed a significant decrease in total OTUs and the Shannon index indices in the early period (up to 4 days) of administration of carbadox in 6-weeks-old piglets, and Looft *et al*.^4^,^27^ also observed significant changes in microbial community structure (*β*-diversity indices) with antibiotic treatment. The discrepancies between the present study and the previous work^4^,^27^,^33^ might be resulted from the use of different type, dosage of antibiotics, the different environmental conditions or pig ages.

*Firmicutes, Tenericutes* and *Bacteroidetes* were the most dominant phyla in this study. These results were similar to previous studies^1^,^4^,^31^ where they showed that *Firmicutes* and *Bacteroidetes* were the most dominant phyla in pig fecal samples. Here we showed that *E. faecalis* UC-100 administration had no significant effect on proportions of dominant phyla. However, on d 28 of feeding, dietary *E. faecalis* UC-100 decreased the abundance of *Fibrobacteres* phyla. *Fibrobacteres* is a small and normal bacterial phylum which benefits the host by fermenting dietary fiber into short-chain fatty acids (SCFAs)^34^. Meanwhile, dietary antibiotics also tend to decrease the bacterial abundance of *Fibrobacteres* on d 28. These results are similar to results of a previous study where the authors showed that weaned pigs treated with tylosin had a lower proportion of *Fibrobacteres* sequences than those in the control group^28^. O’Toole PW *et al*.^35^ reported that consumption of probiotic may modulate the microbiota by competing for nutritional substrates, and by altering the dynamics of carbohydrate utilization by individual microbiota components. It can be speculated that *E. faecalis* UC-100 may modulate the *Fibrobacteres* by competing for nutritional substrates such as cellulose.

*Lactobacillus, Bulleidia* and *Clostridium* were the most predominant genera in the present study. These results are similar to a previous study where they showed that *Lactobacillus* and *Clostridium* were the most dominant genera in pig fecal samples^31^. Although most genera were shared among groups at the same age, 12 genera were uniquely identified in pigs from the *E. faecalis* group on d 14 or 28 of feeding. Of these unique genera, *Anaerovorax* functions to reduce the susceptibility to *Campylobacter* infection in humans^36^, *Deinococcus* is safely used as a feed supplement for hens^37^, and *Paraprevotella* may contribute to host health^38^. Conversely, it was found that *Achromobacte* and *Gemella* were specific to the basal diet group on d 14 and 28 of feeding, and several species of these 2 genera are opportunistic pathogens that affect humans^39^,^40^. In addition, 11 genera including *Veillonella* were uniquely identified in pigs from the antibiotic group on d 14 or 28 of feeding. Some *Veillonella* species have the function of utilization of macro- and micro-nutrients and may contribute to the regulation of host metabolism and body weight in human gut^41^. The existence of the unique beneficial genera in the *E. faecalis* group or the antibiotic group and the unique opportunistic pathogens in basal diet group may be a potential factor related to decreased incidence of diarrhea and increased body weight gain in the *E. faecalis* group and the antibiotic group.

Moreover, it was found that the bacterial abundance of 12 genera were increased as pigs aged in the basal diet group, but decreased in both the *E. faecalis* and antibiotic group on d 28. Of these 12 genera, many species of *Pseudomonas*^42^, *Acholeplasma*^43^, *Arcobacter*^44^,^45^,^46^, and *Eubacterium*^47^ are opportunistic pathogens that affect humans and animals. Several species of *Pseudomonas*^42^ and *Arcobacter*^46^ infections can cause diarrhea. Both antibiotic^1^ and *E. faecalis*^19^,^20^,^21^,^22^,^23^ can reduce or inhibit the presence of opportunistic pathogens, and this may be the reason that the bacterial abundance of these genera decreased and then caused the decreased incidence of diarrhea and the increased body weight gain in both *E. faecalis* UC-100 and antibiotic group. And the microbial shifted in the porcine gut in response to diets fed *E. faecalis* were similar to the response to dietary supplementation of antibiotics, indicating that *E. faecalis* UC-100 could be a potential alternative to the use of antibiotics in pigs to promote health and growth of host.

In addition, the bacterial abundance of 4 genera including *Fibrobacter* and *Megasphaera* were decreased, and *Selenomonas* were increased only in the pigs from the *E. faecalis* group compared with the basal diet group on d 28. A decrease in *Fibrobacter* genus was consistent with the decrease in *Fibrobacteres* phyla in *E. faecalis* group on d 28 of feeding. Some species of *Megasphaera* may cause diarrhea^48^, and *Selenomonas* was related to obesity^49^. The decrease of *Megasphaera* and the increase of *Selenomonas* may cause the decreased incidence of diarrhea and the increased body weight gain in the *E. faecalis* group. Meanwhile, the bacterial abundance of *Bacillus* and *Sphaerochaeta*, were decreased and the bacterial abundance of *Vibrio* and *Zhouia* were increased only in pigs from the antibiotic group compared with the basal diet group. Some species of *Bacillus* may cause cutaneous, gastrointestinal, and inhalation anthrax^50^, its decreased abundance may cause the decreased incidence of diarrhea in the antibiotic group. Previous study^51^ showed that bacteria of *Vibrio* might lead to development of acute gastroenteritis characterized by diarrhea, headache, vomiting, nausea, and abdominal cramps, its increased abundance in the antibiotic group might be due to its resistance to antibiotic^52^.

It was interesting to note that administration of *E. faecalis* UC-100 did not increase the abundance of *Enterococcus* genus. The lack of an effect on *Enterococcus* genera is probably due to the insufficient contribution of the *Enterococcus faecalis* strain, as *E. faecalis* UC-100 after intake still accounted for a minor part of the *Enterococcus* community in our samples.

## Conclusion

The abundance and diversity of the gut microbiota in pigs of the *E. faecalis* group and the bacterial diversity in the antibiotic group were inhibited on d 28 of feeding. Most genera were shared among groups at the same age, 12 and 11 genera were uniquely identified in pigs from the *E. faecalis* group and the antibiotic group on d 14 or 28 of feeding, respectively. Several species of these unique genera can be beneficial to host health. The abundance of *Fibrobacteres* phylum and 12 genera including *Fibrobacter* and some opportunistic pathogens in pigs from both the *E. faecalis* group and the antibiotic group were lower than that in the basal diet group on d 28 of feeding. These results showed that microbial shifts in the porcine gut in response to diets fed *E. faecalis* were similar to the response to dietary supplementation of antibiotics, indicating that *E. faecalis* can be a potential alternative to the use of antibiotics in pigs.

## Materials and Methods

### Probiotics

*E. faecalis* UC-100 (CGMCC No.1.0130) is certified as an animal feed additive by the Ministry of Agriculture in China. The commercial product (the viable count of 1 × 10^10^ CFU/g) was obtained from Sikefu Biotechnology CO., LTD (Beijing, China).

### Animals and sample collection

This experiment was approved by Animal Care and Use Committee of Nanjing Agricultural University. All procedures and the use of animals were carried out in accordance with the Guide for the Care and Use of Laboratory Animals prepared by the Institutional Animal Care and Use Committee of Nanjing Agricultural University, Nanjing, China.

The experimental design and animal feeding procedure have been described previously^25^. Briefly, 150 newly weaned pigs (Duroc × Landrace × Yorkshire, 25 days of age at 8.4 ± 0.2 kg body weight, weaned at day 25) were allotted to 5 dietary treatments based on a randomized complete block design with gender and initial body weight as blocks. Each dietary treatment had 4 pens (replicates), and each pen had 7 or 8 pigs. Dietary treatments represent a basal diet, 3 test diets containing *E. faecalis* UC-100 at various levels (100, 200, and 400 g/t, respectively), or a positive control diet containing multiple antibiotics (bacitracin zinc 40 g/t, aureomycin 75 g/t, and colistin 20 g/t). Dietary treatments were given to pigs for 28 days. All pens were decontaminated and disinfected for 7 days before the pigs moved in to ensure minimal bacterial contamination. The building was temperature-controlled (26.3 ± 2 °C) during the study. Feed and water were available ad libitum for all pigs. Diet composition and nutrient contents are provided in the supplementary material (Table S2). The experiment was divided into 2 phases: phase I (from d 0 to d 14 of feeding) and phase II (from d 14 to d 28 of feeding). Fecal samples were collected from 1 randomly selected pig in all pens by rectal massage on d 0, 14 and 28 of feeding and then stored at −80 °C before DNA extraction. Each group had the same ratio between barrows and gilts. Because 200 g/t *E. faecalis* UC-100 showed the benefits similar to antibiotic supplementation from the previous manuscript^25^, only 36 fecal samples from the *E. faecalis* UC-100 group(200 g/t), the basal diet group and the positive control diet group collected on d 0, 14, and 28 of feeding were used in the current study based on the objective of investing microflora changes in the porcine distal gut in response to the treatment with *E. faecalis* UC-100 as alternatives to antibiotics. Gut microbiota population in fecal samples were assessed through 16 S rRNA gene sequencing.

### DNA extraction, PCR amplification of 16 S rRNA gene, amplicon sequence and sequence data processing

Microbial genomic DNA was extracted from 220 mg of each fecal sample using a TIANamp Stool DNA Kit (Spin Column, Cat. no. DP328) according to the manufacturer’s recommendation. Successful DNA isolation was confirmed by agarose gel electrophoresis^31^.

The V4 hypervariable regions of 16 S rRNA gene were amplified by PCR using the barcoded fusion primers referred to previous study^53^. The primer sequences were 520 F 5-AYTGGGYDTAAAGNG-3 and 802 R 5-TACNVGGGTATCTAATCC-3. The PCR condition was as follows: initial denaturation at 94 °C for 4 min; 94 °C denaturation for 30 s, 50 °C annealing for 45 s, and 72 °C extension for 30 s, repeated for 25 cycles; final extension at 72 °C for 5 min. The PCR amplicon products were separated on 0.8% agarose gels and extracted from the gels. Only PCR products without primer dimers and contaminant bands were used for sequencing by synthesis. Barcoded V4 amplicons were sequenced using the paired-end method by Illumina MiSeq with a 7-cycle index read. Sequences with an average phred score lower than 30, ambiguous bases, homopolymer runs exceeding 6 bp, primer mismatches, or sequence lengths shorter than 100 bp were removed. Only sequences with an overlap longer than 10 bp and without any mismatch were assembled according to their overlap sequence. Reads that could not be assembled were discarded. Barcode and sequencing primers were trimmed from the assembled sequence^31^.

### Taxonomy classification and sequence analysis

Taxon-dependent analysis was conducted using the Greengene database^54^. Greengenes is a quality controlled, comprehensive 16 S reference database and taxonomy based off a *de novo* phylogeny that provides standard operational taxonomic unit sets. OTUs were counted for each sample to express the richness of bacterial species with an identity cutoff of 97%. Low abundance OTUs (fewer than 5 reads) were filtered out of our analysis^55^. The OTU abundance of each sample was generated at genus level. The mean length of all effective bacterial sequences without primers was 227 bp. The abundance count at the genus level was log_2_ transformed and then normalized as follows: from each log-transformed measure, the arithmetic mean of all transformed values was subtracted, and the difference was divided by the standard deviation of all log-transformed values for a given sample. After this procedure, the abundance profiles for all samples exhibited a mean of 0 and a standard deviation of 1.

A Venn diagram was generated to compare OTUs between groups, and the bacterial community indices applied here included Ace and Good’s coverage. The bacterial abundance is shown by Ace. Good’s coverage estimates what percent of the total species is represented in a sample. The bacterial diversity is shown by the number of OTUs. *β*-diversity was calculated using weighted UniFrac distance and displayed using principal coordinate analysis (PCoA)^28^.

### Data analysis

Only high-quality sequences obtained after quality control analysis were used in present analysis which were uploaded to QIIME for further study^54^. All effective bacterial sequences were compared to the Greengene databases using the best hit classification option to classify the abundance count of each taxon. The sequence length was archived by QIIME. The abundance and diversity indices were generated using Mothur with an OTU identity cutoff of 97% after implementing a pseudo-single linkage algorithm^1^. For all parameters, data were compared using a one-way analysis of variance (ANOVA) at the end of each bioassay. A mean comparison was performed using Fisher’s least significant difference test (LSD) and the Duncan multiple range test with a significance level of *P* < 0.05.

## Additional Information

**How to cite this article**: Li, P. *et al*. Microbial shifts in the porcine distal gut in response to diets supplemented with *Enterococcus Faecalis* as alternatives to antibiotics. *Sci. Rep.* **7**, 41395; doi: 10.1038/srep41395 (2017).

**Publisher's note:** Springer Nature remains neutral with regard to jurisdictional claims in published maps and institutional affiliations.

## Supplementary Material

Supporting Information

## Figures and Tables

**Table 1:** Unique genera in the basal diet group, the *Enterococcus faecalis* group and the antibiotic group on d 14 of feeding.

Basal diet group	Antibiotic group	*Enterococcus faecalis* group
*Achromobacter*	*Acetobacter*	*Anaerovorax*
*Ammoniphilus*	*Aequorivita*	*Brevibacterium*
*Azospira*	*Anaerococcus*	*Deinococcus*
*Gemella*	*B-42*	*Facklamia*
*Marinobacter*	*Holdemania*	*Ignatzschineria*
*Natronincola_Anaerovirgula*	*HTCC*	*Mycoplasma*
	*Mycobacterium*	*Pedobacter*
	*Sporanaerobacter*	*Sphingobium*
	*Tsukamurella*	*Vibrio*
	*Veillonella*	

**Table 2:** Unique genera in the basal diet group, the *Enterococcus faecalis* group and the antibiotic group on d 28 of feeding.

Basal diet group	Antibiotic group	*Enterococcus faecalis* group
*Acholeplasma*	*Ramlibacter*	*Burkholderia*
*Achromobacter*		*Paraprevotella*
*Arcobacter*		*Stenotrophomonas*
*Brevundimonas*		
*Comamonas*		
*Coprothermobacter*		
*Desulfotomaculum*		
*Edwardsiella*		
*Facklamia*		
*Gemella*		
*KSA1*		
*Leptolyngbya*		
*Marinobacter*		
*Natronincola_Anaerovirgula*		
*Pseudoramibacter_Eubacterium*		
*Rubrobacter*		
*Tissierella_Soehngenia*		
*Trichococcus*		
*Veillonella*		

**Table 3:** The bacterial abundances of 13 distinct genera were compared among 3 phases (d 0, 14, and 28 of feeding) in the basal diet group.

Genus	d 0	d 14	d 28
*Acholeplasma*	0.00 ± 0.00^b^	0.00 ± 0.00^b^	1.25 ± 1.25^a^
*Arcobacter*	0.25 ± 0.25^B^	0.00 ± 0.00^B^	1.00 ± 0.71 ^A^
*Caldicoprobacter*	0.00 ± 0.00^b^	0.00 ± 0.00^b^	17.75 ± 16.09^a^
*Desulfotomaculum*	0.00 ± 0.00^b^	0.00 ± 0.00^b^	1.25 ± 1.25^a^
*Fibrobacter*	0.75 ± 0.75^B^	3.25 ± 1.11^B^	21.50 ± 10.43 ^A^
*Ignatzschineria*	0.00 ± 0.00^b^	0.00 ± 0.00^b^	22.25 ± 22.25^a^
*KSA1*	0.00 ± 0.00^b^	0.00 ± 0.00^b^	3.75 ± 3.75^a^
*Leptolyngbya*	0.00 ± 0.00^b^	0.00 ± 0.00^b^	1.25 ± 1.25^a^
*Natronincola_Anaerovirgula*	0.00 ± 0.00^b^	0.25 ± 0.25 ^ab^	1.25 ± 1.25^a^
*Pseudomonas*	1.25 ± 1.25^b^	2.00 ± 0.71^b^	45.00 ± 41.01^a^
*Pseudoramibacter_Eubacterium*	0.25 ± 0.25^b^	0.00 ± 0.00^b^	4.25 ± 3.92^a^
*Tepidimicrobium*	0.25 ± 0.25^b^	0.00 ± 0.00^b^	31.25 ± 29.60^a^
*Tissierella_Soehngenia*	0.00 ± 0.00^b^	0.00 ± 0.00^b^	1.25 ± 1.25^a^

**Table 4:** The bacterial abundances of 12 distinct genera were compared among the basal diet group, the *Enterococcus faecalis* group and the antibiotic group on d 28 of feeding, and these genera shifts caused by the *Enterococcus faecalis* group was similar with the antibiotic group.

Genus	Basal diet group	Antibiotic group	*Enterococcus faecalis* group
*Acholeplasma*	1.25 ± 1.25^a^	0.00 ± 0.00^b^	0.00 ± 0.00^b^
*Arcobacter*	1.00 ± 0.71 ^A^	0.00 ± 0.00^B^	0.00 ± 0.00^B^
*Caldicoprobacter*	17.75 ± 16.09^a^	0.75 ± 0.25^b^	0.75 ± 0.48^b^
*Desulfotomaculum*	1.25 ± 1.25^a^	0.00 ± 0.00^b^	0.00 ± 0.00^b^
*Ignatzschineria*	22.25 ± 22.25^a^	0.50 ± 0.29^b^	0.00 ± 0.00^b^
*KSA1*	3.75 ± 3.75^a^	0.00 ± 0.00^b^	0.00 ± 0.00^b^
*Leptolyngbya*	1.25 ± 1.25^a^	0.00 ± 0.00^b^	0.00 ± 0.00^b^
*Natronincola_Anaerovirgula*	1.25 ± 1.25^a^	0.00 ± 0.00^b^	0.00 ± 0.00^b^
*Pseudomonas*	45.00 ± 41.01^a^	2.25 ± 0.75^b^	2.25 ± 0.25^b^
*Pseudoramibacter_Eubacterium*	4.25 ± 3.92^a^	0.00 ± 0.00^b^	0.00 ± 0.00^b^
*Tepidimicrobium*	31.25 ± 29.60^a^	1.00 ± 0.41^b^	2.00 ± 1.08^b^
*Tissierella_Soehngenia*	1.25 ± 1.25^a^	0.00 ± 0.00^b^	0.00 ± 0.00^b^

**Table 5:** The bacterial abundances of 9 distinct genera were compared among the basal diet group, the *Enterococcus faecalis* group and the antibiotic group on d 28 of feeding, whereas these genera shifts caused by the *Enterococcus faecalis* group was different with the antibiotic group.

Genus	Basal diet group	Antibiotic group	*Enterococcus faecalis* group
*Anaerococcus*	2.75 ± 2.75^a^	0.25 ± 0.25^ab^	0.00 ± 0.00^b^
*Bacillus*	4.75 ± 2.06^a^	1.25 ± 0.95^b^	2.00 ± 1.68^ab^
*Fibrobacter*	21.50 ± 10.43 ^A^	8.75 ± 6.43 ^AB^	3.00 ± 0.41^B^
*Megasphaera*	73.00 ± 42.18^a^	31.00 ± 14.36^ab^	18.00 ± 9.49^b^
*Selenomonas*	0.00 ± 0.00^b^	0.25 ± 0.25^ab^	0.75 ± 0.25^a^
*Sharpea*	29.50 ± 26.51^a^	382.50 ± 379.83^ab^	1.00 ± 0.41^b^
*Sphaerochaeta*	14.50 ± 5.56^a^	2.50 ± 0.65^b^	6.50 ± 5.20^ab^
*Vibrio*	0.00 ± 0.00^b^	2.00 ± 2.00^a^	0.25 ± 0.25^ab^
*Zhouia*	0.00 ± 0.00^B^	1.75 ± 0.85 ^A^	0.75 ± 0.75^AB^
