# Supplementary material for: Microbial shifts in the porcine distal gut in response to diets supplemented with Enterococcus Faecalis as alternatives to antibiotics
Source: Sci Rep. 2017 Feb 6;7:41395. doi: 10.1038/srep41395 (PMC5292720; doi:10.1038/srep41395)
Supplement: Supporting Information [file srep41395-s1.pdf]

Microbial shifts in the porcine distal gut in response to diets supplemented with *Enterococcus Faecalis* as alternatives to antibiotics

Pinghua Li [1,2*], Qing Niu [1,2*], Qingtian Wei [1,2], Yeqiu Zhang [1,2], Xiang Ma [1,2], Sung Woo Kim [3], Mingxin Lin[4], Ruihua Huang [1,2+]

[1] Institute of Swine Science, Nanjing Agricultural University, Nanjing, 210095, China, [2] Huaian Academy of Nanjing Agricultural University, Huaian, 223005, China, [3] Department of Animal Science, North Carolina State University, Raleigh, North Carolina, 27695, United States of America, [4] Changxing Ecoagriculture Co. Limited, Yixing, 214246, China

[+]Corresponding author: Ruihua Huang. Institute of Swine Science, Nanjing Agricultural University, Nanjing 210095, China. Tel: +86-25-84395362; +86-13814540789; Fax: +86-25-84395314; E-mail: Ruihua Huang, rhhuang@njau.edu.cn.

* These authors contributed equally to this work.

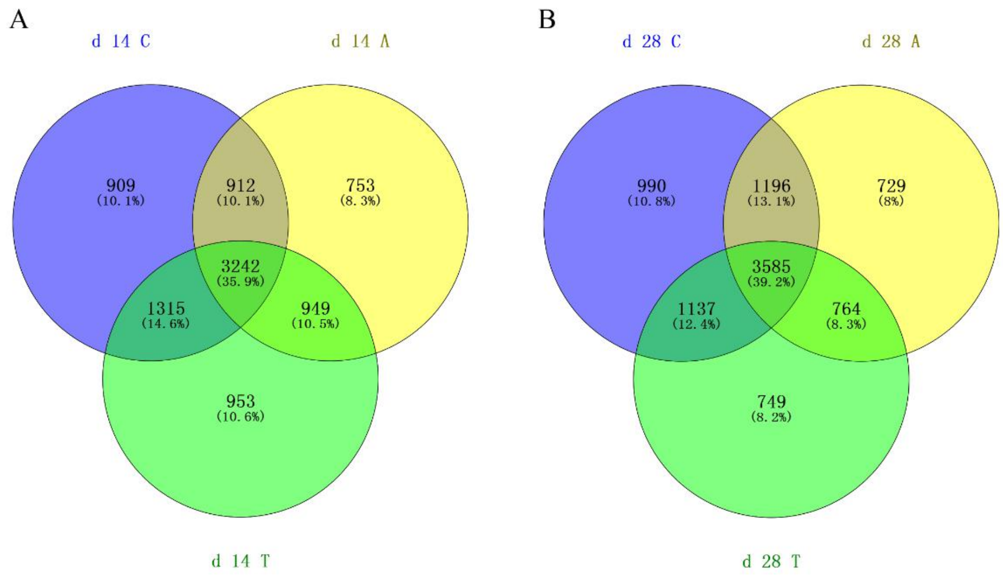

Fig.S1 A Venn diagram of OTUs in different groups. A Venn diagram was generated to compare OTUs between the 3 groups (C represents the basal diet group, A represents the antibiotic group, and T represents the *Enterococcus faecalis* group) at the same time points [d 14 (A) and d 28 (B)] and to depict OTUs that were unique to different groups.

A

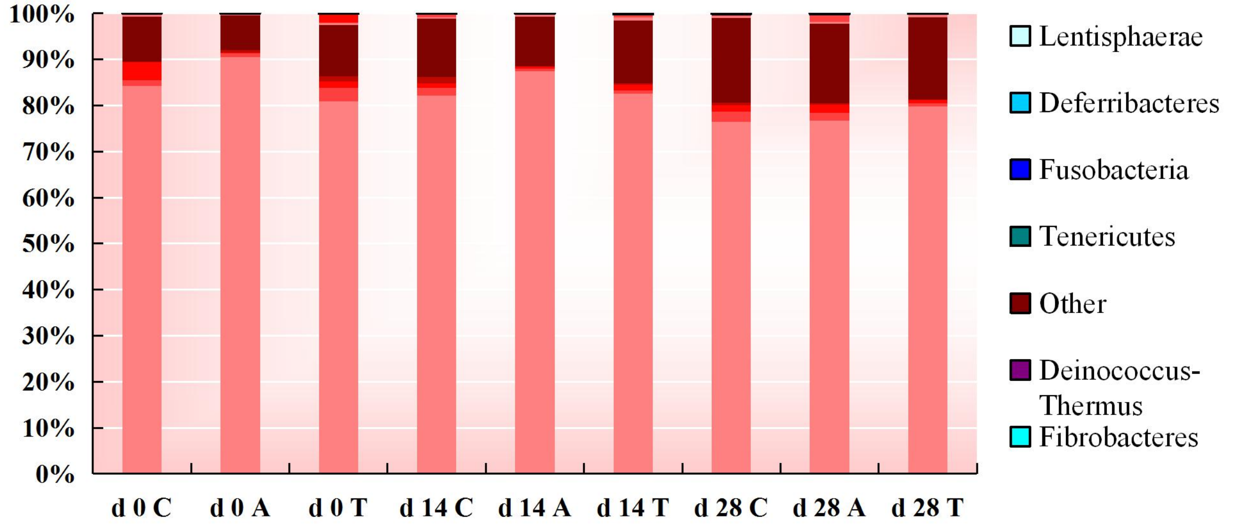

B

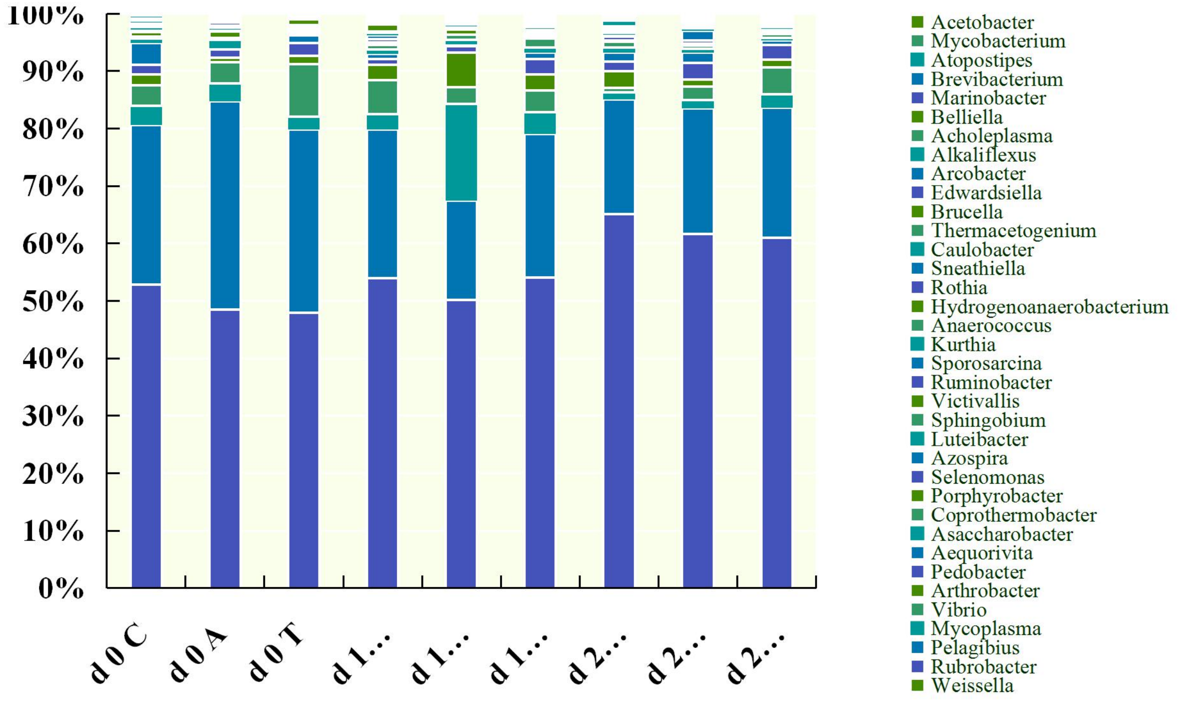

Fig. S2 Biological classification distribution of gut microbiota. Distribution of the phyla (A) and genera (B) as a percentage of the total number of identified 16S rRNA gene sequences in individual groups. C represents the basal diet group, A represents the antibiotic group, and T represents the *Enterococcus faecalis* group.

Table S1 Raw reads, read length, sequences and OTUs from nine groups.

Groups	d 0				d 14				d 28			
	Reads	Read length	High quality sequences	Modified OTUs	Reads	Read length	High quality sequences	Modified OTUs	Reads	Read length	High quality sequences	Modified OTUs
Basal diet group	201,303	0.21G	194,242	6,073	212,206	0.22G	204,687	6,378	222,669	0.23G	213,274	6,908
Antibiotic group	196,387	0.20G	190,619	5,446	207,015	0.21G	200,588	5,856	215,410	0.22G	208,099	6,274
Enterococcus faecalis group	222,402	0.23G	215,053	6,032	217,627	0.23G	210,558	6,459	216,349	0.22G	209,635	6,235

Table S2 Composition and nutrient levels of the basal diet (as-feed basis, %).

A

**Ingredient**	**Content**
Corn	60
Soybean meal	23
Bean oil	2
Concentrates 1)	15
Total	100

B

**Nutrient levels 2)**	**Content**
DE (MJ/kg) 2)	13.9
CP	18.9
EE	3.4
CF	4.3
Crude Ash	6.0
Ca	0.9
TP	0.65

1) Nutritional composition concentrates(% or Content per kg): CP 30%, Lys3.0%，VA 70 KIU，VD 13 KIU，VE 160 IU，Fe 1000 mg ，Cu 1000 mg，Zn 650 mg，Ca 5%，P 2%，NaCl 2.5%.

2) DE was a calculated value, while the others were measured values.
